# Neuroplasticity and the next wave of antidepressant strategies

**DOI:** 10.3389/fncel.2013.00218

**Published:** 2013-11-20

**Authors:** Shawn Hayley, Darcy Litteljohn

**Affiliations:** Department of Neuroscience, Carleton UniversityOttawa, ON, Canada

**Keywords:** depression, relapse, ketamine, erythropoietin, combined treatment, neurogenesis, BDNF

## Abstract

Depression is a common chronic psychiatric disorder that is also often co-morbid with numerous neurological and immune diseases. Accumulating evidence indicates that disturbances of neuroplasticity occur with depression, including reductions of hippocampal neurogenesis and cortical synaptogenesis. Improper trophic support stemming from stressor-induced reductions of growth factors, most notably brain derived neurotrophic factor (BDNF), likely drives such aberrant neuroplasticity. We posit that psychological and immune stressors can interact upon a vulnerable genetic background to promote depression by disturbing BDNF and neuroplastic processes. Furthermore, the chronic and commonly relapsing nature of depression is suggested to stem from “faulty wiring” of emotional circuits driven by neuroplastic aberrations. The present review considers depression in such terms and attempts to integrate the available evidence indicating that the efficacy of current and “next wave” antidepressant treatments, whether used alone or in combination, is at least partially tied to their ability to modulate neuroplasticity. We particularly focus on the N-methyl-D-aspartate (NMDA) antagonist, ketamine, which already has well documented rapid antidepressant effects, and the trophic cytokine, erythropoietin (EPO), which we propose as a potential adjunctive antidepressant agent.

This special issue entitled “Neuroimmune modulation for brain plasticity and repair” essentially deals with how immune and trophic factors differentially influence pathological brain situations, with a view towards informing the development of novel disease-modifying therapies. In particular, whereas certain key growth factors such as brain-derived neurotrophic factor (BDNF) have pro-neuroplastic and neuroprotective actions, pro-inflammatory stimuli (particularly certain cytokines) exert predominantly anti-neuroplastic and pro-death effects. This relationship appears to hold across a range of central conditions, including major depression, anxiety disorders, Parkinson’s disease (PD), Alzheimer’s disease (AD), multiple sclerosis (MS), and traumatic brain injuries. Immune-based strategies aimed at limiting the actions of pro-inflammatory factors and/or stimulating those of key growth factors may therefore hold particular promise for treating chronic brain disease.

In this review article we focus specifically on recent advances in the treatment of depressive illness, exploring in detail how trophic mediators, modulators and mechanisms may be relevant for antidepressant action. Depression is particularly germane to this special issue given not only its strong link with trophic/inflammatory factors, but also the fact that the disorder is highly co-morbid with most neurological diseases. Indeed, one-third to one-half of all PD patients has major depression (Rojo et al., 2003; Yamanishi et al., 2013), and a similar proportion of AD and MS patients likewise suffers from the condition (Holtzer et al., 2005; Siegert and Abernethy, 2005). It is very likely that such high rates of co-morbid depressive symptomology are at least partially attributable to the common pro-inflammatory state evident in these brain conditions. In fact, during MS relapses (in the most common relapsing-remitting form of the disease) when pro-inflammatory levels are highest, depressive symptoms also tend to peak (Kahl et al., 2002; Koutsouraki et al., 2011). Moreover, in PD patients, retrospective studies indicated that signs of depression were often evident years before the onset of motor symptoms or PD diagnosis (Jacob et al., 2010). Hence, depression in at least a subset of PD cases probably stems from antecedent pathological changes (during the so-called prodromal period) occurring most likely outside of the basal ganglia motor circuitry (Litteljohn et al., 2010). There is in this regard a growing recognition that, in the PD brain, Lewy body pathology occurs early—and probably even starts—in brainstem and limbic nuclei (Goedert et al., 2013). As is the case for senile plaques in AD, these *α*-synuclein-containing inclusion bodies, which are hallmark signs of PD, are almost universally associated with robust microglial activation and elevated levels of inflammatory cytokines (Reynolds et al., 2008; Phani et al., 2012). Thus, early-occurring pro-inflammatory changes might be one common mechanism accounting for co-morbid depressive and potentially other neuropsychiatric symptoms across a range of neurological disease states.

It could be argued that an enhanced neuroinflammatory tone stemming from primary disease pathology or possibly even psychosocial stressor exposure might set the stage for depression by causing alterations in the function or abundance of key trophic factors implicated in normal neuroplastic and pro-survival events (or by simply overtaxing them). Indeed, depressive behaviors provoked by interferon-*α* (IFN-*α*) and interleukin-1*β* (IL-1*β*) were associated with reduced BDNF levels and reductions of hippocampal neurogenesis, and these effects normalized upon administration of the IL-1*β* receptor antagonist, IL-1ra (Anisman et al., 2008; Dedoni et al., 2012). Similarly, treatment with the bacterial endotoxin, lipopolysaccharide (LPS), or the viral mimic, polyinosinic-polycytidylic acid (polyI:C), augmented central (hippocampus, frontal cortex) and peripheral pro-inflammatory cytokine concentrations whilst reducing BDNF levels, and these effects coincided with pronounced memory deficits (Kranjac et al., 2012) and depressive-like behaviors (Gibney et al., 2013). In fact, infection with live influenza virus induced changes analogous to those provoked by the LPS and polyI:C challenges (Jurgens et al., 2012), and a recent study by Ji et al. (2011) likewise implicated disturbed pro-inflammatory cytokine-neurotrophin crosstalk in the cognitive impairment following chronic amyloid-*β* treatment in mice.

Secondary to or at least facilitated by such inflammatory-driven perturbations of trophic signaling, inadequate or improper neural connections could conceivably be recruited and engaged to deal with the “wear and tear” of life’s stressors. If one takes the point of view that depression stems, at least in part, from faulty wiring of emotional and fear sensitive circuitry within the brain, then it stands to reason that finding the means to “re-wire” such circuitry and maintain these changes is the key to addressing the fundamental biological basis for the condition. Hence, using cognitive behavioral and other psychotherapeutic methods in addition to pharmacological treatments that directly target biological processes linked to faulty neural wiring may be essential for adequate treatment.

Consistent with the theme of this special issue, it is our contention that immune- and- stressor-induced changes in neuroplasticity, involving adult neurogenesis, synaptogenesis, dendritic remodeling, and trophic signaling, are ultimately responsible for the biological manifestations of the “faulty wiring” posited to occur in depression. The present review will target two key aspects of this hypothesis. Firstly, we will review the data in favor of a neuroplastic-trophic hypothesis for depression and integrate them with new evidence indicating that traditional monoamine acting drugs act through neuroplastic processes to provoke therapeutic effects. Secondly, we explore in detail novel emerging treatments for depression that may more directly target neuroplastic circuits and act to at least temporarily “re-wire” neural circuits at the systems levels. As an example, emerging evidence suggests that certain agents with novel antidepressant properties, such as ketamine, might modify the connectivity of diverse cortical circuitry involved in the generation of consciousness, sense of self and potentially rumination. We will also introduce the possibility that certain immune cytokines that have trophic properties [e.g., erythropoietin (EPO)] might contribute novel antidepressant properties consistent with a neuroplastic view of depression.

## Neuroplasticity and depression: a role for existing antidepressants

### Neuroplasticity and rumination

Neuroplastic changes at the molecular and cellular level must eventually come to reverberate through neural circuits at the systems level. Indeed, meaningful changes in behavior, thought patterns and emotions are complex and require concerted communication between multiple brain regions. In this regard, the recent breakthrough in our understanding of the basic neuronal circuitry that gives rise to our “default” or “resting state” has caused a substantial paradigm shift in how we view consciousness, self-referential thinking (introspection) and ruminative processes. The so-called default mode network (DMN) comprises a series of interconnected cortical brain regions that are highly active during restful or un-challenged states (e.g., insula, cingulate, frontal and parietal regions). However, during goal- and- task-oriented activity, when specific thalamo-cortical pathways are engaged to appropriately deal with the task at hand, the DMN regions de-activate or reduce their metabolic activity (Raichle et al., 2001). Interestingly, this task-associated shifting between DMN rest state and alternative activation pathways appears to be disrupted in depressed patients (Sheline et al., 2009; Sliz and Hayley, 2012). Specifically, such individuals fail to appropriately down-regulate DMN activity and, hence, get “stuck” in self-focused states and have difficulty smoothly shifting to the required task. This is likely the neural machinery that contributes to the increased negative ruminations evident in depression.

It is possible that disturbances of neuroplasticity within DMN brain regions could contribute to faulty reverberations of this circuit, which with repeated activation would only serve to strengthen negative ruminations in depressed individuals. However, a very recent study offers hope that antidepressant drug treatments might target the DMN to help “re-wire” faulty circuitry. Specifically, Scheidegger et al. (2012) reported that ketamine treatment reduced DMN metabolic activity and diminished DMN connectivity within the cingulate and prefrontal cortices, albeit in healthy non-depressed participants. One could easily imagine that such an effect of ketamine and potentially other antidepressant drugs could help give depressed patients the needed “push” to move them out of a current negative ruminative state, and enable them to derive benefit from completing external tasks or otherwise focusing on or engaging with positive environmental stimuli. Ultimately, such positive interactions would be expected to help foster or strengthen appropriate emotional neural connections and promote synaptogenesis (as has been reported with ketamine treatment) in brain regions adversely affected by the stress and negative thinking associated with depression.

The importance of rumination in the depressive response must not be underestimated, and the sad fact is that excessive rumination over one’s negative plight and ways to “fix” the situation actually likely strengthens the very depressiogenic pathways that give rise in the first place to the low mood and host of other depressive symptoms. Hence, a better understanding of how neuroplastic changes at a cellular level translate into reverberations of systems that influence and/or underpin rumination is key to finding more effective and rapid means of ameliorating depression, as well as its co-morbid anxiety symptoms. Although cognitive behavioral techniques for learning how to break out of ruminative cycles are undoubtedly of substantial importance, when in the throes of depression such strategies may seem entirely untenable and are therefore perhaps more useful during continuation and maintenance stages to prevent relapse once the illness is more under control. To this end, ketamine and the development of similar rapid neuroplastic modulator drugs could be just the solution to the ruminative crisis.

### Neuroplasticity, depression and relapse

An important but often overlooked aspect of depression is the high degree of relapse that occurs even in individuals that initially responded well to antidepressant drugs. As such, some enduring neural changes might underlie the presumed pathological brain circuits. In this regard, considerable evidence has supported the contention that *protracted* disturbances of neuroplasticity might occur in depression. In particular, major depression is associated with both structural and functional changes within discrete brain regions, including the hippocampus, amygdala and prefrontal cortex (PFC; Drevets et al., 2008; Sacher et al., 2012). Particular attention has focused on the reduced hippocampal volume often observed in patients diagnosed with major depression, and post-mortem as well as brain imaging analyses indicated that the extent of the hippocampal reduction was related to illness duration (Bremner et al., 2000; MacQueen et al., 2003; Colla et al., 2007). Recent evidence has highlighted the possibility that persistent alterations of neuroplasticity result in faulty communication between anterior cingulate cortex (ACC), PFC, hippocampal, and amygdaloid regions–thus giving rise to disturbed processing of emotionally salient information (Schlösser et al., 2008; Carballedo et al., 2011).

A particularly important point to consider is the fact that the reductions in regional brain volume that are evident in depression can be effectively reversed with successful treatment to remission (Banasr et al., 2011; Arnone et al., 2012). Thus, structural anomalies in depression need not necessarily be permanent, underscoring the importance of early intervention to stave off enduring and potentially even progressive brain damage. The possibility has been entertained that reductions of neurogenesis and their correction with antidepressant treatment might be one mechanism accounting for hippocampal volume variations (Malberg, 2004; Boldrini et al., 2009). Of course, the relatively low number of new neurogenic cells normally produced in adulthood suggests that this is not the only process involved [the exciting recent discovery by Spalding et al. (2013) that a full third of hippocampal neurons are subject to exchange across the human lifespan strongly hints, however, at an important functional role of adult neurogenesis in health and disease]. Other findings have indicated that changes in glial cell density and the complexity of dendritic arbors might also account for volumetric changes in depression (Tata and Anderson, 2010; Gittins and Harrison, 2011). Indeed, reductions of cortical and/or hippocampal astrocytes were reported in depressed patients (Rajkowska et al., 1999; Rajkowska, 2000, as well as in stressor-based animal models of the disease (Banasr et al., 2010; Liu et al., 2011). Stressors also have well known inhibitory actions on dendritic branching (e.g., Son et al., 2012), raising the possibility that grey matter shrinkage may be related at least in part to the considerable stress experienced by depressed patients. In fact, Hercher et al. (2010) reported that depressed patients who committed suicide displayed reduced dendritic length of pyramidal neurons in the ACC.

These different scenarios are not mutually exclusive; whatever the case, it is still unclear as to whether regional brain volume reductions are causally implicated in depression or arise from some secondary aspect of the illness. At the very least, grey and/or white matter volumetric changes provide a useful biomarker for the structural state of the brain and potentially the duration of depressive illness (Cheng et al., 2010; Arnone et al., 2012). Further, the degree of volume reduction could possibly provide information regarding the likelihood of positive treatment responses and, conversely, the risk of relapse. Future studies are needed to assess if and how such structural changes map onto the probability of relapse, especially since it is easy to envision that volumetric reductions could give rise to faulty processing of emotional stimuli, as well as potentially contribute to ruminations.

In addition to reductions of neuroplasticity, it should be underscored that *increased* neuroplasticity could, in certain cases, contribute to depressive symptomology and relapse. This is analogous to the case of addictive behaviors, wherein “negative” plasticity represents a re-wiring of hedonic and craving pathways. This point is perhaps best illustrated in the case of the amygdala, which displays increased dendritic arborization in response to stressors rather than the reductions observed in the PFC and hippocampus (Vyas et al., 2002). Stressors were likewise reported to increase BDNF levels in the amygdala whilst reducing them in the hippocampus (Lakshminarasimhan and Chattarji, 2012). Similarly, while BDNF had antidepressant-like effects when administered directly into the hippocampus (Shirayama et al., 2002; Ye et al., 2011), it actually provoked depressive-like behaviors when infused into the ventral tegmental area (VTA) (the source of dopaminergic innervation of the PFC and limbic nuclei) (Eisch et al., 2003). Enhanced plasticity of amygdaloid nuclei could increase vulnerability to depression by virtue of augmented “fear” or threat processing. In fact, BDNF signaling is known to be required for amygdala-dependent fear learning in rodents (Rattiner et al., 2004; Ou and Gean, 2006). This would be especially important in the context of relapse given that depressed individuals in remission often display heightened vigilance and arousal; even modest stressors could engender small relapses that if not immediately dealt with could progress to full-fledged relapse. It may be that enhanced negative plasticity persists in the VTA or amygdala among some (or all) individuals with a history of depression, and underlies these individuals’ heightened vulnerability to relapse.

### Neurotrophic factors, depression and antidepressant responses

Several reports have indicated that platelet and serum BDNF protein concentrations are suppressed in depressed subjects, with levels of the growth factor correlating with symptom severity (Pandey et al., 2010; Yoshida et al., 2012). BDNF mRNA expression was similarly decreased in leukocytes of depressed patients, and treatment with the selective serotonin reuptake inhibitor (SSRI), escitalopram, normalized this deficit (Cattaneo et al., 2010). Moreover, in clinical populations, patient improvement coincided with plasma and serum BDNF returning to normal levels (Piccinni et al., 2008; Teixeira et al., 2010). Interestingly, BDNF in circulating lymphocytes was even suggested as a possible biomarker to predict antidepressant treatment response (venlafaxine) (Rojas et al., 2011).

Animal models have extended the human findings to include impaired hippocampal neurogenesis and aberrant neuronal morphology in the discussion of neurotrophic and neuroplastic mechanisms in depression. Indeed, impaired hippocampal neurogenesis was evident in rodents exposed to a chronic corticosterone or stressor regimen (McEwen, 2005; Diniz et al., 2013), and hippocampal implantation of cortisol pellets among vervet monkeys induced irregular cell layers, soma shrinkage and dendritic atrophy (Sapolsky et al., 1990). Likewise, in subordinate male tree shrews, psychosocial stress caused a reduction in the complexity of apical dendrites (both dendrite length and number of branch points) on pyramidal hippocampal and PFC neurons (Magariños et al., 1996). Importantly, stressor exposure in animals (using a wide variety of stressor preparations) has been routinely found to alter central BDNF protein and/or mRNA levels, and these BDNF changes are considered to be instrumental for the negative effects of stressors on neurogenesis and neuronal morphology and cytoarchitecture (Masi and Brovedani, 2011).

Besides their potentiating effects on circulating BDNF, several clinically beneficial therapies, including SSRIs, tricyclics, and electroconvulsive therapy (ECT), were reported to augment hippocampal BDNF expression (Castrén et al., 2007). In fact, it would appear that the vast majority of known effective antidepressant treatments stimulate BDNF and affect neurogenesis. For instance, all SSRIs and tricyclic antidepressants, as well as new alternate treatments such as vagal stimulation and deep brain stimulation (DBS), positively influence hippocampal neurogenesis (Castrén and Rantamäki, 2010; Encinas et al., 2011; Yan et al., 2011). Similarly, aerobic exercise and enriched environments typically increase hippocampal BDNF, and their antidepressant-like effects in the context of a stressor depend upon intact neurogenesis (Ernst et al., 2006; Schloesser et al., 2010). Furthermore, direct brain infusion of BDNF (considered a neurotrophic cytokine itself) promoted sprouting of central serotonin (5-HT) neurons (Mamounas et al., 1995) and, when administered directly into the hippocampus, the neurotrophin produced antidepressant-like behavioral effects (Shirayama et al., 2002).

Despite the rather strong evidence linking antidepressant drug responses to BDNF, recent work has uncovered several notable exceptions. For instance, the tricyclic desipramine did not affect PFC or hippocampal BDNF, and escitalopram actually decreased BDNF levels in these brain regions (Jacobsen and Mørk, 2004). Further, while one week of escitalopram increased BDNF mRNA, three weeks led to a reduction of transcript levels in the hippocampus (Alboni et al., 2010). These findings clearly contrast with other studies demonstrating antidepressant drug-induced elevations of BDNF both in the basal state and in response to chronic stress (Balu et al., 2008; Zhang et al., 2010). Some of the discrepancies between findings in BDNF studies might be related to the fact that the protein is enzymatically processed in such a complex manner. Indeed, there are believed to be nine different promoters controlling BDNF transcription and it has been predicted that there may be 22 different BDNF mRNA isoforms (Zheng et al., 2012). There is also evidence to suggest that BDNF protein and mRNA follow distinct temporal patterns of induction upon antidepressant administration. For instance, Musazzi et al. (2009) showed that BDNF protein levels were increased in the hippocampus and PFC after 1–2 weeks of reboxetine treatment, whereas the BDNF mRNA elevation only became apparent after 3 weeks. A further complicating issue is the fact that typical antidepressant treatments appear unable to influence BDNF release in an activity-dependent manner. This could help explain the characteristic time lag in therapeutic action of many of these drugs, given that rapid antidepressant actions have been linked to a rapid BDNF response (Duman et al., 2012). Ultimately, differences in the timing of exposure and the nature of the antidepressant or other eliciting stimulus likely uniquely influence the post-translational processing of BDNF, as well as the transcriptional machinery recruited to the BDNF promoter and other regulatory regions.

Although BDNF has undoubtedly received the most attention, emerging and recent evidence indicates that other growth factors, most notably glial cell-line derived neurotrophic factor (GDNF) (a member of the transforming growth factor beta family) is also likely involved in depression. Indeed, GDNF protein and mRNA levels were diminished in the blood of depressed patients (especially in later life) (Takebayashi et al., 2006; Otsuki et al., 2008; Diniz et al., 2012; Tseng et al., 2013), and successful antidepressant treatment (e.g., with SSRIs, ECT) caused a return to normal levels (Zhang et al., 2008, 2009). Yet, Wang et al. (2011a) found that higher levels of plasma GDNF correlated with cognitive impairment in late-onset depression, and Michel et al. (2008) reported finding increased GDNF concentrations in the parietal cortex among autopsied depressed patients. Thus, similar to the case of BDNF, it appears that the relationship between GDNF and depression may be more nuanced and complex than perhaps initially thought, and compensatory processes are likely involved.

Nonetheless, in rodents, antidepressants were found to not only normalize stressor-induced reductions in circulating GDNF (Angelucci et al., 2003) but also to induce the neurotrophin’s synthesis and release from cultured glioma cells and astrocytes (Mercier et al., 2004; Golan et al., 2011; Di Benedetto et al., 2012). Similarly, hippocampal GDNF levels were markedly diminished in rats exposed to chronic stress, and clomipramine treatment (a tricyclic agent) reversed this deficit whilst ameliorating the stressor-induced behavioral symptoms (Liu et al., 2012). Interestingly, Uchida et al. (2011) recently reported that chronic stress caused changes in histone modification and DNA methylation of the GDNF gene promoter within the mouse ventral striatum. What’s more, these epigenetic modifications proved to be critical in determining susceptibility (or resistance) to the depressive- and- anxiety-like behavioral effects of the stressor (Uchida et al., 2011). In light of these findings and given the critical role ascribed to GDNF in the development and function of hippocampal cells, it is possible that alterations of GDNF are relevant for the “plasticity deficit” that appears in depression. Indeed, continuous infusion of GDNF into the striatum markedly increased cell proliferation and neurogenesis in the adult canine hippocampal dentate gyrus (Chen et al., 2005). And more recently, Kohl et al. (2012) showed that chronic fluoxetine treatment robustly stimulated adult hippocampal neurogenesis among *α*-synculein-overexpressing mice (which display profound basal impairments in neurogenesis) via the induction of hippocampal BDNF and GDNF.

### Poly-treatment approaches in depression

It has become clear that poly-drug treatment strategies often lead to a greater likelihood of remission and reduce the risk of depressive relapse (Blier et al., 2010; El Mansari et al., 2010). This is not altogether surprising given the wide spectrum of physical and emotional symptoms typically experienced by patients. Indeed, the neurovegetative symptoms of depression, which include disturbed sleep and feeding, as well as agitation and psychomotor retardation, are clearly rooted in neural pathways that are distinct from those subserving the cognitive and emotional aspects of the disease (e.g., melancholia, anhedonia). A polypharmacy approach in depression thus allows for the tailoring of treatment to the individual (i.e., based on the specific symptom clusters exhibited by each patient). For instance, augmentation of SSRI and serotonin-norepinephrine reuptake inhibitor (SNRI) treatments with low-dose atypical antipsychotics (e.g., risperidone, aripiprazole) has been shown to not only boost the basic antidepressant response on mood (especially among treatment-resistant and/or suicidal patients) but also to greatly diminish anxiety and neurovegetative symptoms (Reeves et al., 2008; Trivedi et al., 2008; Blier and Blondeau, 2011; Chen et al., 2011; Owenby et al., 2011). At the same time, combining SSRIs with atypical antidepressants such as mirtazapine also greatly alleviates certain neurovegatative features, particularly disturbed sleep (Holm and Markham, 1999; Blier et al., 2009; Jindal, 2009). Ultimately, the heterogeneity in both symptom profile and response profile to the various SSRIs probably stems from the complexity of genetic backgrounds in depression, as well as wide variation in depressed patients’ history of prior stress.

Besides having the advantage of affecting—at times synergistically—multiple different mood-relevant neuro­trans­mitter systems (e.g., 5-HT, norepinephrine (NE) and dopamine (DA); El Mansari et al., 2010; Masana et al., 2012), evidence is beginning to suggest that multi-drug treatments may induce more robust, rapid and/or long-lasting neuroplastic changes than single-drug treatments. For instance, imipramine-plus-metyrapone (a glucocorticoid synthesis inhibitor), which showed therapeutic promise in animal models and a treatment-resistant human sample, caused a synergistic increase in BDNF mRNA expression within rat hippocampus and cortex (Rogóz and Legutko, 2005). More recently, augmentation of fluoxetine with the 5-HT2A receptor antagonist, ketanserin, induced a more potent increase of hippocampal BDNF mRNA and *β*-catenin protein (which is important for cellular proliferation and differentiation during development and repair) than either drug alone, and these effects coincided with diminished forced swim immobility (Pilar-Cuéllar et al., 2012). Similarly, (Marchetti et al. (2010) demonstrated that subchronic combinatorial imipramine and rolipram (a phosphodiesterase type 4 inhibitor) synergistically reduced immobility in the forced swim test (FST) and potentiated activity-dependent transcription in the hippocampus. Moreover, the combination treatment augmented glutamatergic transmission in hippocampal CA1 pyramidal neurons and increased the dendritic spine density of these cells; long-term potentiation at CA1 synapses was also enhanced Marchetti et al. (2010). And finally, using a chronic stress rat model of depression, Xu et al. (2006) revealed that the atypical antipsychotic, quetiapine, and the SNRI, venlafaxine, acted synergistically and at relatively low doses to prevent the stressor-induced reductions in hippocampal neurogenesis and BDNF expression. Thus, it is likely that the heightened therapeutic response engendered by multi-drug preparations stems, at least in part, from the additive and synergistic effects of these treatments on a range of cellular and molecular neuroplastic processes.

Evidence is also beginning to suggest that atypical and second-generation antidepressants, aside from being useful as “add-ons” in augmentation therapy (or as later-line but sometimes even first-line monotherapies), could be helpful in alleviating some of the side effects induced by traditional antidepressant medicines [see Bauer et al. (2013) for a good recent review of the major side effects associated with typical and newer generation antidepressants]. For instance, Ozmenler et al. (2008) reported that mirtazapine augmentation in remitted depressed patients with SSRI treatment-emergent sexual dysfunction led to a significant reduction of depressive symptoms and, in nearly half the cases, completely ameliorated the SSRI-induced sexual problems. More generally, it was suggested that combination antidepressant therapy might allow for lower doses of the separate component drugs to be used (relative to monotherapy), which could improve patient tolerability and adherence to treatment (Goodwin et al., 2009). Yet, it should be underscored that many of the prospective and accepted adjunctive agents, particularly the atypical antipsychotics, are themselves associated with potentially severe adverse effects (Nelson and Papakostas, 2009; Bauer et al., 2013). Moreover, a polypharmacy approach to treating depression is necessarily associated with an increased risk of drug-drug interactions; this is especially so among individuals already taking multiple medications—older persons and HIV-infected patients receiving antiretroviral therapy, for instance (Hill and Lee, 2013).

Another strategy for treating depression that has garnered substantial clinical and research interest is the combining of pharmacological and psychotherapeutic modalities (usually interpersonal or cognitive behavioral therapy; CBT). Over the years there have been numerous studies supporting the clinical effectiveness of such an approach, especially in cases of chronic major depression (de Maat et al., 2007). Recently for instance, Köhler et al. (2013) reported that CBT augmentation markedly improved clinical outcomes among acutely depressed patients receiving pharmacological treatment in a naturalistic psychiatric setting. Moreover, a large-scale randomized controlled trial of the effectiveness of CBT augmentation to pharmacotherapy in treatment-resistant depression yielded strikingly positive results (Wiles et al., 2013). Data pointing to a beneficial effect of antidepressants-plus-psychotherapy in the initiation and/or continuation phases of treatment likewise exist for child and adolescent populations (Kennard et al., 2008; Lynch et al., 2011), as well as for depressed geriatric patients (Hollon et al., 2005). It is somewhat surprising then that several recent meta-analyses failed to provide compelling evidence (either sparse or none at all) in support of combined psychotherapy and pharmacotherapy in depression (Imel et al., 2008; Cox et al., 2012; Cuijpers et al., 2012; Jakobsen et al., 2012; von Wolff et al., 2012). However, it should be noted that most if not all of these meta-analyses were limited by the small number of published randomized controlled trials meeting inclusion criteria (limited data availability), as well as the relatively small sample sizes used in the included reports (limited power).

Importantly, just as pharmacological treatments have been shown to normalize regional brain functional abnormalities in depression (e.g., Mayberg et al., 2000; Heller et al., 2013), so too have several of the more common psychological therapies used for treating the disease (e.g., CBT). Even long-term psychodynamic interventions were linked to persistent neurobiological changes in depressed patients (Buchheim et al., 2012). In a recent systematic review on the subject, Quidé et al. (2012) set forth compelling evidence that both pharmacological and psychological treatments normalize functional (and in the former, structural too) alterations of a wide-ranging “fear network” in the depressed and anxious brain, and that these common ends are most likely achieved through divergent means. Specifically, whereas antidepressant drugs tend to decrease limbic hyperactivity (and, consequently, emotional reactivity), “talk therapy” appears to work by strengthening, building and restoring frontal cortex capacity (i.e., for self-referential processing and emotional regulation), particularly in the ACC and medial PFC (Ritchey et al., 2011; Quidé et al., 2012; Yoshimura et al., 2013). Clearly, distinct yet convergent neuroplastic mechanisms are likely involved in the antidepressant action of pharmacological (primarily “bottom-up”) and psychological (more “top-down”) therapies, and there is a potential, therefore, for additive or even synergistic cross-modal antidepressant effects. This could be relevant not only for treatment initiation but also relapse prevention and maintenance of the antidepressant response over time.

Although a considerable degree of success has been had in recent years using combinatorial treatments of existing antidepressant treatments, there remains a desperate need for the development of novel pharmacological, genetic and other agents. This is especially fuelled by the significant number of treatment-resistant patients (only some of whom respond to combination therapy with extant treatments), as well as the recent surge in experimental and clinical studies demonstrating that, besides monoaminergic and hormonal alterations, reductions in trophic factors (particularly BDNF) together with fundamental disturbances in various elements of neuroplasticity might contribute to the development and evolution of depression. To this end, we now turn our attention to emerging evidence demonstrating the potential antidepressant efficacy of two novel agents, ketamine and EPO—both of which appear to greatly affect neuroplastic processes.

## Novel antidepressant treatments to target neuroplasticity from a cellular to systems level: a focus on ketamine and erythropoietin

The search for novel antidepressants with targets outside of the monoaminergic pathways has gained momentum in recent years. Indeed, evidence has begun to reveal that aside from the typical 5-HT, NE and DA circuits, multiple other systems appear to be affected in depression. There is, for instance, a growing recognition that stress neuropeptide systems, most notably the corticotropin-releasing hormone (CRH) signaling network, are dysregulated in depression, and recent attempts have been made to develop antidepressant agents that antagonize the actions of CRH and its related peptides (Holsboer and Ising, 2010). Non-pharmacological depression treatments that influence neuroplasticity have also begun to emerge; these include DBS (as a late-line option in severe cases), transmagnetic stimulation (TMS) and vagal stimulation. Of course, ECT has long been considered a potent inducer of neuroplasticity (though fraught with potentially serious side effects), and psychotherapeutic modalities too are beginning to be conceptualized in terms of their neuroplastic potential (Beauregard, 2009). The substantial range of physiological, biochemical and psychological targets that one would expect these different treatments to impact speaks to the complexity of pathways and mechanisms that are likely affected in depression.

### Ketamine and depression

Exciting emerging evidence indicates that the non-competitive N-methyl-D-aspartate (NMDA) glutamate receptor antagonist, ketamine, which is widely used for its anesthetic and analgesic properties, shows promise as a novel treatment for depression. Indeed, a single injection of very low-dose (subanesthetic) ketamine was found not only to promote fast-acting (within hours) antidepressant and anti-suicidal effects but also to be effective in heretofore treatment-resistant patients (Berman et al., 2000; Zarate et al., 2006; Liebrenz et al., 2007; Price et al., 2009; DiazGranados et al., 2010; Ibrahim et al., 2011). Furthermore, the antidepressant effects following a single ketamine dose have been reported to persist for several days to even weeks (Correll and Futter, 2006; Liebrenz et al., 2007; Irwin and Iglewicz, 2010; Mathew et al., 2010). Hence, ketamine is particularly unique in its clinical profile, making it of potentially enormous therapeutic significance. However, the safety of long-term treatment in depressed patients has yet to be fully evaluated, although there are indications that chronic low-dose ketamine may be tolerable, feasible and effective (Liebrenz et al., 2009; aan het Rot et al., 2010; Messer et al., 2010; Murrough et al., 2013). Moreover, despite some important recent advances in our understanding of the various molecular and cellular sequelae following low-dose ketamine treatment (see below), the precise mechanisms underlying ketamine’s antidepressant effects are not fully known. Emerging and future studies examining ketamine’s mode(s) of action therefore hold the potential to shed much-needed new light on the pathological underpinnings of depression. This, in turn, would be expected to lead to more specific therapeutic targets and the development of safer and more effective drugs.

The rapid antidepressant response following ketamine treatment, which contrasts sharply with the long antidepressant time lag of several weeks-to-months that is characteristic of traditional monoamine acting drugs (e.g., SSRIs), may at least partially be related to the drug’s direct neuronal effects in the PFC and hippocampus (Duman et al., 2012). Consistently, we recently found that a single intraperitoneal injection of racemic ketamine (5 mg/kg), which comprises equal (50:50) concentrations of the (R)- and (S)-enantiomers of ketamine (the latter is roughly 4 times more active, possesses better pharmacokinetic properties and is potentially more tolerable than the former) (Paul et al., 2009; Mion and Villevieille, 2013), altered the levels both of 5-HT and its metabolite, 5-HIAA, within the PFC (Clarke and Hayley, unpublished findings). Interestingly, although ketamine increased PFC 5-HT concentrations in the basal state, it prevented the changes in serotonergic neurotransmission following acute restraint stress (Clarke and Hayley, unpublished findings). Others have likewise reported that the antidepressant-like effects of ketamine were dependent upon an intact 5-HT system. Indeed, 5-HT depletion using para-chlorophenylalanine blocked the ability of a single injection of ketamine to reduce immobility in a FST (Gigliucci et al., 2013). However, the nature of the behavioral effects of ketamine and the extent of 5-HT involvement in them may vary with a number of factors, including dose, number of treatments and time of testing relative to drug administration. For example, Chindo et al. (2012) found that forced swim immobility was actually enhanced (and not reduced) 24 h after the final of five daily ketamine injections (30 mg/kg/day). Moreover, the 5-HT acting antidepressant, paroxetine, as well as the atypical antipsychotics, clozapine and risperidone, reversed the ketamine-enhanced immobility (Chindo et al., 2012). Although temporal variation in 5-HT functioning could account for the observed differences in forced swim immobility following ketamine treatment, recruitment over time of alternate neurotransmitter systems might also play a role. Indeed, chronic ketamine markedly up-regulated midbrain DA synthesis and levels, together with midbrain BDNF concentrations (Tan et al., 2012). In fact, not only can ketamine influence brain glutamatergic and monaminergic systems (serotonin, DA and NE), but ketamine is also capable of modulating the activity of GABAergic, cholinergic, opioidergic, and even purinergic circuits—albeit at doses considerably higher than those typically employed in the clinical and preclinical depression studies (Mion and Villevieille, 2013).

**Figure 1:**
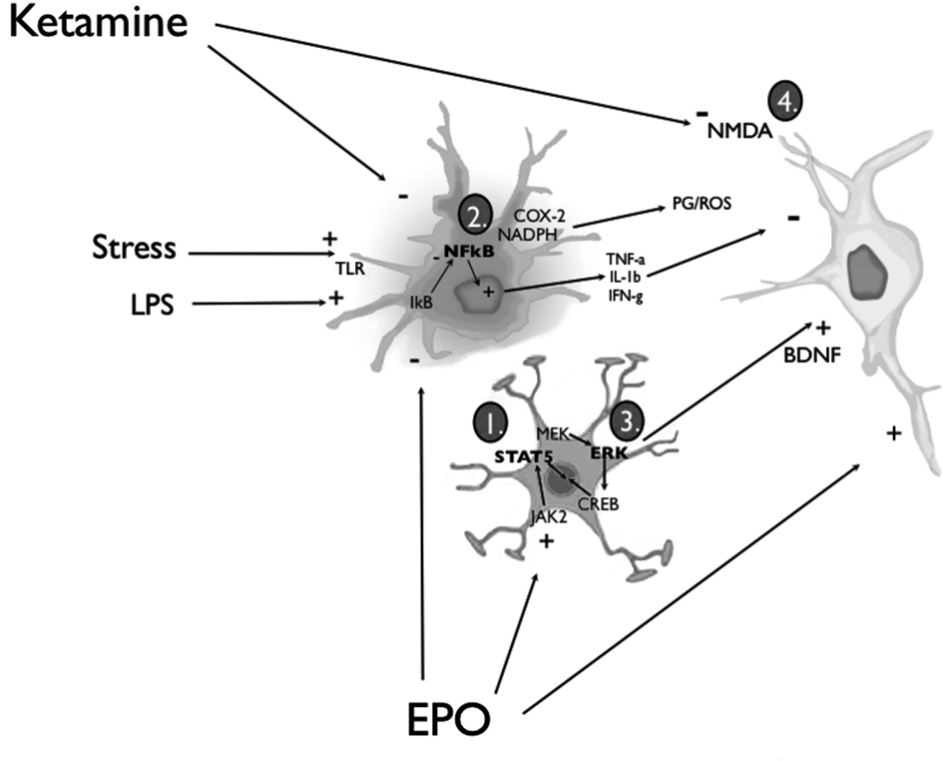
**Simplified diagram of the routes through which EPO or ketamine might affect glia and neurons to promote anti-depressant-like consequences**. EPO can act upon astrocytes and neurons by (1) inducing the activation of JAK/STAT and PI3K/Akt signal transduction pathways or (3) promoting MAP kinase-MEK signaling, culminating in extracellular signal-regulated kinase (ERK) phosphorylation and the recruitment of cyclic adenosine monophosphate (cAMP) response element-binding protein (CREB). Engagement of these EPO/EPOR signaling pathways effectively biases the activity of pro- and anti-apoptotic cascades towards the latter, and increases the synthesis and release of BDNF from activated astrocytes and neurons. Signaling in neurons through its TrkB receptors (and the multiple associated signal transduction cascades), BDNF can promote a wide range of neuroplastic changes (e.g., enhanced synthesis of synaptic proteins and neurotrophins) that ultimately favor cell survival. In parallel, EPO can exert anti-inflammatory actions by (2) inhibiting the liberation of nuclear factor-*κ*B (NF-*κ*B) from its inhibitory binding partner, IkB, in microglial cells. Thus, pro-inflammatory cytokine release (e.g., tumor necrosis factor-*α* (TNF-*α*), IL-1*β* and IFN-*γ*), as well as prostaglandin (PG) and reactive oxygen species (ROS) production via COX-2 and NADPH oxidase enzymes is inhibited. EPO can also cross the BBB to directly interact with neuronal receptors. Ketamine was similarly found to be capable of (2) attenuating the activation of microglia and astrocytes, as well as a variety of peripheral immune cells; the end result, once again, is an overall dampening of potentially neurodestructive pro-inflammatory responses. Nonetheless, a majority of the studies investigating the antidepressant-like action of ketamine have focused on the downstream molecular sequelae of the drug’s NMDA glutamate receptor antagonism (4). Considered to be of paramount importance in this regard are the up-regulation of BDNF (e.g., via inhibition of eEF2 kinase) and the activation of synaptogenic signaling pathways (i.e., mTOR/p70S6 kinase) (see text for additional details).

In addition to alterations of classic neurotransmitter systems, much attention has been afforded the possibility that rapid BDNF changes contribute importantly to the fast-acting antidepressant effects of ketamine (see Figure 1). In this regard, Autry et al. (2011) reported that NMDA receptor blockade by ketamine or MK801 (dizocilpine), which is similar in action to ketamine but who’s neurotoxic consequences preclude its use clinically, rapidly increased BDNF levels in mouse hippocampus secondary to the deactivation of eukaryotic elongation factor-2 (eEF2) kinase (which normally suppresses the translation of BDNF by phosphorylating eEF2). Furthermore, the swift rise in hippocampal BDNF coincided with reduced immobility in the FST, and conditional BDNF knockout completely abrogated ketamine’s (as well as MK801’s) antidepressant-like behavioral effects (Autry et al., 2011). Similarly, the antidepressant efficacy of ketamine was reduced among depressed patients carrying the loss-of-function BDNF Met66 alleles (compared to the more common Val/Val genotype), as well as in stressed rodents bearing the human BDNF Val66Met polymorphism (Laje et al., 2012; Liu et al., 2012). And finally, treatments that enhanced the antidepressant effects of ketamine (e.g., the analgesic agent, tramadol) were also found to rapidly (within 1 h of treatment) up-regulate hippocampal BDNF, along with its TrkB receptor (Yang et al., 2012). Importantly, then, variation in the *speed* of the neuroplastic responses induced by different treatment modalities may be one potential mechanism accounting for the stark temporal differences in the antidepressant action of ketamine and more conventional antidepressant agents. Indeed, whereas ketamine elevated hippocampal BDNF concentrations within 30 min of administration (Autry et al., 2011), SSRI and tricyclic antidepressants increased central BDNF levels only after several days or weeks of treatment (Martínez-Turrillas et al., 2005; Larsen et al., 2008).

Interestingly, ketamine was also observed to induce robust alterations of PFC synapse structure and function, including enhanced dendritic branching and synaptic receptor number and density (e.g., GluR1-containing AMPA receptors) (Li et al., 2010). These changes, which are consistent with increased synaptogenesis and neuronal synaptic plasticity, followed a rapid time course (within 24 h of ketamine) only slightly longer than that seen with the BDNF changes. In fact, the neuroplastic effects of ketamine have been posited to stem from the ability of ketamine to expeditiously stimulate the mTOR signaling pathway, most likely via a BDNF-dependent mechanism; the resultant activation of ribosomal p70S6 kinase is considered to drive the rapid protein translation and fast synaptogenic changes that are evident following acute ketamine treatment (Duman et al., 2012). Indeed, Li et al. (2010) demonstrated that the mTOR pathway is rapidly activated following low-dose ketamine, with increases in PFC synaptic signaling proteins (e.g., PSD-95, GluR1) and dendritic spines following shortly after. Moreover, the synaptogenic and antidepressant behavioral effects of ketamine were completely abolished in rats treated with the mTOR inhibitor, rapamycin (Li et al., 2010, 2011). These findings raise the possibility that ketamine could affect mood by rapidly enhancing plastic excitatory transmitter signaling and the processing of BDNF. In fact, a very recent study indicated that, in treatment-resistant depressed patients, acute ketamine augmented plasma BDNF levels and induced electroencephalographic (EEG) changes consistent with enhanced synaptic strength and plasticity (e.g., increased early sleep slow wave activity and high-amplitude waves, increased slow wave slope) (Duncan et al., 2013). Moreover, the BDNF changes varied directly with the EEG parameter changes, and only those individuals positively responding to the ketamine treatment displayed this link (Duncan et al., 2013).

At the same time, SSRI agents too have been associated with synaptic “re-wiring” and “re-modeling”. For instance, citalopram partially attenuated the reduction in hippocampal dendritic spine density following dim light-at-night stress in hamsters (Bedrosian et al., 2012), and fluoxetine enhanced dendritic spine density and altered glutamatergic receptor stoichiometry in rat forebrain (Ampuero et al., 2010). Such changes normally are characterized by a substantial time delay and usually coincide with the onset of a positive clinical response (Ampuero et al., 2010). Similarly, the hippocampal volume reductions evident in depressed patients usually normalize after the patients go into remission or at least show substantial improvement (Banasr et al., 2011). Hence, the actual structural brain changes that are induced by the various antidepressant treatments (albeit differentially) might be a crucial common feature in bringing about a positive clinical effect. Indeed, Liu et al. (2012) recently reported that homozygous BDNF Met/Met mice, besides showing a reduced antidepressant response to acute ketamine, were completely insensitive to the PFC synaptogenic effects of the drug.

### Ketamine and neuroimmune factors

Ketamine possesses potent and rapid anti-inflammatory effects that could conceivably be relevant for its antidepressant actions. Numerous studies have demonstrated that ketamine inhibits the production of pro-inflammatory cytokines, including IL-1*β* and TNF-*α*, as well as the expression of NF-*κ*B (an important inflammatory transcription factor), following immunological challenge with LPS (Takenaka et al., 1994; Sakai et al., 2000; Sun et al., 2004; DeClue et al., 2008). More recent reports have indicated that ketamine, besides reducing the activation of peripheral antigen presenting cells (e.g., macrophages and dendritic cells) and the consequent elaboration of inflammatory mediators, is capable of inhibiting LPS-induced microglial activation, at least in part by negatively regulating MAP kinase activity (Chang et al., 2009; see Figure 1). Consistently, Yang et al. (2013) found that ketamine administration in rats attenuated the LPS-induced rise in PFC IL-6 and IL-1*β*, as well as the reduction in IL-10; importantly, these immunoregulatory effects coincided with the amelioration of LPS-induced depressive-like behavior in the FST. Additionally, ketamine has been demonstrated to attenuate NF-*κ*B activation and the expression of the pathogen-associated molecular recognition receptor, Toll like receptor-4 (TLR-4), in astrocytes (Wu et al., 2012). Taken together, these data clearly show that ketamine can influence several aspects of peripheral and central immune signaling, including the initiation of the inflammatory response (e.g., TLR-4), activity and/or levels of downstream effectors (e.g., IL-1*β* and TNF-*α*), and recruitment of intra-cellular messenger pathways (e.g., NF-*κ*B and MAP kinases). Importantly, each of these immune mechanisms has been implicated in clinical depression and, to some extent, various neurodegenerative conditions.

It has recently been posited that low-grade cerebral inflammation could predispose individuals to depression and suicide by disturbing glutamate neurotransmission (Erhardt et al., 2013). Indeed, increased colony stimulating factor (CSF) levels of the NMDA agonist, quinolinic acid (QUIN), as well as the inflammatory cytokine, IL-6, were reported in suicide victims, and there was a positive correlation between IL-6 and QUIN in suicide attempters (Erhardt et al., 2013). Moreover, an increased density of QUIN-expressing microglia was evident in suicidal individuals (Steiner et al., 2008, 2011). Others have shown that cerebral infections also elevate QUIN together with pro-inflammatory cytokines (Heyes et al., 1995). Thus, ketamine could have beneficial effects in depressed patients, and in particular among individuals who are suicidal or at an increased risk of suicide (e.g., suicide attempt survivors, treatment-resistant patients), by countering the QUIN elevation (via its NMDA antagonism) and, at the same time, inhibiting IL-6 and other inflammatory mediators. In this regard, ketamine completely prevented the hippocampal neuronal damage and neurodegeneration following intracerebroventricular QUIN adminstration in rats (Henschke et al., 1993).

### Emerging glutamatergic modulators and combination treatment strategies

Despite the mounting evidence indicating that ketamine has rapid and robust antidepressant properties (and notwithstanding the earlier mentioned preliminary clinical data indicating that long-term, low-dose ketamine may be both tolerable and effective; e.g., Messer et al., 2010), concerns over ketamine’s psychotomimetic effects have spurred intensive efforts to develop safer and more tolerable glutamate-based antidepressants. At the vanguard of this movement are the “next generation” NMDA receptor antagonists. Included here are the aminoadamantanes, memantine and amantadine (Sani et al., 2012); the NR2B-selective antagonists, traxoprodil (CP-101,606; Preskorn et al., 2008) and MK-0657 (Ibrahim et al., 2012a); and the low-affinity NMDA channel blocker AZD6765 (Zarate et al., 2013). The NMDA receptor glycine-site functional partial agonist, GLYX-13, and its orally bioavailable and presumed more potent analog, NRX-1074, have also garnered the recent attention of researchers and clinicians (Burgdorf et al., 2013; Dolgin, 2013), as have several modulators of metabotropic glutamate receptors (e.g., the mGluR7 allosteric agonist AMN082; Bradley et al., 2012) and select *α*-Amino-3-hydroxy-5-methyl-4-iso­xazolepropionic acid (AMPA) receptor potentiators (e.g., Org 26576; Nations et al., 2012). A thorough examination of these up-and-coming glutamatergic modulators falls outside the purview of this review, and we instead refer the interested reader to the excellent recent reviews by Lapidus et al. (2013) and Pilc et al. (2013). Still, it bears mentioning at this juncture that, with the exception of the aminoadamantanes (inconclusive clinical results) and for now NRX-1074 (paucity of published data; Phase I trial underway), very promising preclinical and, where applicable, initial clinical findings have been reported for virtually all of the aforementioned potential new glutamate-based antidepressants.

Even as work is progressing on the development, characterization and testing of these novel glutamatergic agents, optimizing the tolerability and response durability of ketamine-based depression therapies remains an active area of research. In the clinical realm, such efforts have so far been focused largely on identifying add-on or substitution strategies for successfully maintaining the initial rapid antidepressant response induced by ketamine (and thus preventing relapse) (Krystal et al., 2013). Specifically, two small-scale trials have been published to-date examining the efficacy of the glutamatergic modulator, riluzole (which is used to prolong survival in Amytrophic Lateral Sclerosis), in delaying relapse among ketamine-remitted patients with treatment-resistant depression (Mathew et al., 2010; Ibrahim et al., 2012b). Unfortunately, neither of these studies generated positive results; however, as pointed out by Krystal et al. (2013), both trials suffered from a lack of power.

Also garnering recent research and clinical attention has been the idea that combined treatment with ketamine and ECT might result in synergistic enhancements of mood in patients with severe and intractable depression symptoms. Unlike the ketamine-plus-riluzole maintenance therapy trials, this area of research—which was spurred on by a couple of promising case reports (reviewed in Loo et al. (2010))—is concerned not so much with extending the antidepressant effect of ketamine but rather exploring the potential of low-dose ketamine to augment ECT. While the handful of prospective and retrospective clinical studies that have since been published on the subject—their methodological differences notwithstanding—have tended to produce positive results (Okamoto et al., 2010; Kranaster et al., 2011; Loo et al., 2012; Wang et al., 2012), the findings are by no means definitive (e.g., Abdallah et al., 2012; Järventausta et al., 2013) and more testing and optimizing of combined ketamine and ECT in depression is clearly in order.

Apart from these burgeoning clinical efforts, numerous preclinical infrahuman investigations have provided support for the idea that sub-threshold doses of ketamine (with respect to treating depressive-like pathology) could be combined with down-titrated doses of other putative antidepressant agents to promote robust antidepressant-like outcomes, without the attendant psychotomimetic concerns. For instance, it has been more than 10 years since Chaturvedi et al. (1999, 2001) first demonstrated that ketamine displayed antidepressant-like synergism with fluvoxamine and imipramine in the rodent FST. The latter effect was recently recapitulated by Réus et al. (2011), who further showed that combined ketamine-plus-imipramine resulted in a significantly more robust increase of hippocampal, PFC and amygdalar CREB and BDNF levels than each treatment alone. Similarly, combinations of sub-threshold doses of ketamine and lithium synergistically reduced immobility in the rodent FST (Ghasemi et al., 2010; Liu et al., 2013) and enhanced structural and functional neuroplastic changes in the PFC (increased mTOR signaling, dendritic spine density and diameter, and excitatory postsynaptic currents; Liu et al., 2013). Potentiation of ketamine-induced antidepressant-like effects has also been reported with analgesic and anesthetic agents, wherein facilitation of neurotrophin (Yang et al., 2012) and AMPA receptor-mediated signaling Wang et al. (2011b) were found to be of vital importance. Moreover, Akinfiresoye and Tizabi (2013) observed that repeated exposures to a combination of ketamine and AMPA, at doses that were ineffective on their own, induced marked synaptogenic and antidepressant-like effects (increased p-mTOR, synapsin-1 and BDNF; decreased FST immobility). While these preclinical findings have yet to be translated into the clinical realm, it is tempting to at least speculate on the potential role of low-dose “ketamine-plus” combination therapies in depression.

### Erythropoietin (EPO)

Emerging data, albeit relatively scant, indicate that EPO too has the potential to improve the cognitive and emotional symptoms of major depression. In fact, a recent review of the EPO literature demonstrated that four out of an existing five stressor-based animal studies and all seven of the extant human studies (in depressed and non-depressed subjects alike) demonstrated positive effects of EPO with respect to hippocampal-dependent memory and emotion-related behavior (Miskowiak et al., 2012).

EPO is a cytokine that is produced predominately by the kidney. Besides directing the trafficking of immune cells and having anti-apoptotic actions, EPO was recently reported to induce antidepressant-like effects in the forced swim and novelty-induced hypophagia tests (Girgenti et al., 2009). EPO and its receptors (EPOR) are expressed centrally throughout the lifespan, but are particularly abundant in the developing and ageing brain (Sanchez et al., 2009). Moreover, within the hypothalamus, hippocampus and neocortex of normally developing rats, EPO and/or EPOR immunoreactivity was localized primarily to neurons (and not astrocytes or microglia) (Sanchez et al., 2009). Similarly, EPO and EPOR were detected in adult midbrain DA and cortical neurons, as well as cultured cortical and cerebellular neurons (Csete et al., 2004). Yet, astrocytes, microglia and endothelial cells have also been found to express EPO and its receptor, especially following injury or neurodegeneration (Bernaudin et al., 1999; Assaraf et al., 2007; Nadam et al., 2007). Taken together with the fact that the non-hematopoietic carbamylated form of EPO, c-EPO, also has robust CNS effects (Leconte et al., 2011; Ding et al., 2013), it is obvious that EPO has important central effects independent of its impact on red blood cells. Perhaps most importantly with regards to depression and other stress-related disorders, EPO was reported to induce BDNF expression and have potent neuroplastic effects (Leconte et al., 2011; Mengozzi et al., 2012).

As already mentioned, augmented BDNF signaling has been strongly linked to antidepressant outcomes; however, a complication of using BDNF itself clinically is that the neurotrophin does not appreciably cross the blood brain barrier (BBB; Pardridge et al., 1994). BDNF treatment may also be associated with substantial side effects, including those related to pain pathways (Pezet and McMahon, 2006). In contrast, EPO is routinely and safely used to treat anemia (Sargin et al., 2010) and is considered to readily cross the BBB (Brines et al., 2000). While it is true that a few studies failed to detect increased EPO CSF levels with systemic administration (Juul et al., 1997, 1999), others reliably found EPO to be elevated in the CSF of humans and animals after therapeutic systemic doses (Alafaci et al., 2000; Ehrenreich et al., 2002). Indeed, both the murine and human forms of EPO, as well as the human analog often used clinically, decarbepoetin-*α*, all crossed the BBB in untreated naïve mice and accumulated at clinically significant concentrations (Banks et al., 2004).

Given the data implicating hippocampal disturbances in depression, it is particularly significant that EPO protects hippocampal neurons from stressor-induced apoptosis (Kumral et al., 2005; Zhang et al., 2007) and increases adult hippocampal neurogenesis (Wang et al., 2004; Chen et al., 2007; Leconte et al., 2011). At the same time, in rodents, EPO was reported to improve spatial memory in the Morris water maze (Hengemihle et al., 1996; Zhang et al., 2009) and to prevent ischemia-induced cognitive impairments in a passive avoidance task (Sakanaka et al., 1998). Similarly, EPO promoted improved cognitive functioning in MS patients (Ehrenreich et al., 2007a) and among individuals with schizophrenia (Ehrenreich et al., 2007b). Thus, it would appear that EPO has potent cognitive-enhancing actions which seem likely to be at least partially related to the trophic cytokine’s hippocampal neuroplastic effects. Interestingly, recent pre-clinical data also suggest that EPO may hold promise as an agent to promote neuronal recovery. In fact, as pointed out by Sargin et al. (2010), neuroprotective outcomes have been reported in an exceedingly large majority of the nearly 200 studies using EPO in animal models of stroke and traumatic brain injury.

### EPO signaling and depression

Although little evidence to date indicates that central EPO signaling is affected by antidepressant medicines, Girgenti et al. (2009) found that EPO levels were elevated in the hippocampal dentate gyrus after electroconvulsive seizure in rats. As well, in human imaging studies, EPO modulated brain responses to emotional information in both healthy volunteers and depressed patients (Miskowiak et al., 2007, 2009, 2010), just as conventional SSRIs were reported to do (Harmer et al., 2006; Murphy et al., 2009). Similarly, we recently found that systemic EPO treatment had antidepressant-like effects in the FST and blunted the impact of stressor exposure on exploration in open field and elevated plus maze paradigms (Osborn et al., 2013). And consistent with the aforementioned earlier reports documenting the neurogenic potential of EPO (e.g., Leconte et al., 2011), in our hands the hematopoietic growth factor significantly increased hippocampal neurogenesis, and this effect was apparent in naïve and stressed mice alike (Osborn et al., 2013).

As in the case of ketamine, it is critically important to understand the signaling mechanisms through which EPO could impart its antidepressant effects. Briefly, EPO binding promotes the homodimerization of two EPOR molecules, leading to a conformational change that induces the phosphorylation of the receptor-associated Janus kinase-2 (JAK2) protein tyrosine kinase and subsequent activation of intermediate intracellular factors; these include phosphatidylinositide 3-kinase (PI3-K), Akt/protein kinase-B, mitogen-activated protein kinases (MAPKs), and signal transducer and activator of transcription-5 (STAT5; Broxmeyer, 2013; see Figure 1). In non-erythroid cells, EPO acts through a receptor that is also linked to CD131, the common beta chain receptor subunit through which granulocyte-macrophage colony stimulating factor (GM-CSF) also signals (Broxmeyer, 2013). This receptor complex is expressed both by neurons and immune cells, and its stimulation induces STAT5 along with c-Jun N-terminal kinase (JNK), PI3K and MAPK (Brines and Cerami, 2008). Importantly, these signaling cascades have all been linked to the activation of anti-apoptotic factors, cell differentiation, cellular growth, and the modulation of plasticity. For instance, Byts et al. (2008) reported that the induction of STAT5 and Akt in hippocampal neurons was essential for the neurotrophic effects of EPO.

Recent data pointing to a connection between EPO and BDNF may be particularly telling of the mechanisms subserving the potential antidepressant action of EPO. Several reports have indicated that EPO can increase BDNF levels and synthesis (Wang et al., 2004; Girgenti et al., 2009; Mengozzi et al., 2012); this of course contrasts with the known BDNF-antagonizing effects of stressors. It is thus tempting to speculate that an initial EPO-induced rise in BDNF could synergize with further exogenously applied EPO to reinforce specific molecular and cellular mechanisms of antidepressant action. This possibility holds for the prospective combination of EPO with any number of established BDNF-stimulating treatments (e.g., SSRIs, ketamine, and even exercise). Indeed, BDNF and EPO share common intra-cellular signaling pathways, including the PI3-K and MAP kinase cascades. There is, therefore, ample opportunity for the convergence of EPO and BDNF signaling, and studies are definitely warranted to flesh out this possibility (Figure 1).

In contrast to the positive interactions observed between BDNF and EPO, pro-inflammatory stimuli generally act to down-regulate EPO. Indeed, LPS, as well as IL-1*β* and TNF-*α*, were demonstrated to reduce circulatory EPO mRNA and protein levels through mechanisms dependent on NF-*κ*B activation (Nairz et al., 2012). Conversely, EPO itself appears to have potent anti-inflammatory properties, as evidenced by its successful application in a number of chronic inflammatory conditions and/or their animal models (e.g., colitis, MS and diabetes) (Yuan et al., 2008; Nairz et al., 2011; Meng et al., 2013). Likewise, EPO was shown to attenuate or prevent a broad range of central and peripheral pathology following LPS exposure, including lung and brain injury, renal dysfunction, and vascular hypo-reactivity (Kumral et al., 2007; Mitra et al., 2007; di Villa Bianca et al., 2009; Shang et al., 2009); however, several conflicting reports do exist (e.g., Wilms et al., 2009; Wu et al., 2010).

Although very few studies have explicitly investigated how EPO might promote anti-inflammatory responses, a recent report suggests that inhibition of the NF-*κ*B p65 subunit is likely to be essential (Nairz et al., 2011). Importantly, NF-*κ*B signaling is considered the primary pathway mediating the effects of IL-1*β* and TNF-*α* on immune and neural cells (Kataoka, 2009), and a recent study implicated NF-*κ*B as a vital contributor to the anti-neurogenic and depressive-like behavioral consequences of chronic mild stress (Koo et al., 2010). These data raise the intriguing possibility that EPO could impart antidepressant-like effects not only through the stimulation of trophic and neuroplastic processes but also by modulating the inflammatory milieu (by way of restraining inflammatory NF-*κ*B signaling) that is both evident and pathologically relevant in many stressor-related disorders.

## Conclusions

In many cases, the current most efficacious treatment involves the combined administration of more than one antidepressant or other compounds, possibly by promoting synergistic neuronal effects. This reinforces the view that depression involves a spectrum of varied symptoms and likely various etiological mechanisms are involved. In this regard, we posit that stress and dysregulated immune factors acting against a backdrop of genetic vulnerability ultimately shape the evolution of depression by affecting classic neurotransmitter and peptide circuits, as well as interfering with neuroplastic processes.

A sizable proportion of individuals are either totally unresponsive or partially responsive to existing antidepressant treatments. Moreover, patients that do respond positively to antidepressants typically show only partial symptom remission and often relapse after treatment discontinuation, promoting the view that depression is a lifelong condition. The chronic course for the illness suggests that depression could involve persistent neural changes stemming from disturbances of neuroplasticity. Hence, novel strategies that directly target such disturbances, rather than simply managing downstream neurotransmitters, are urgently required.

Novel treatments include the NMDA antagonist, ketamine, which promotes unusually rapid and sustained antidepressant responses after a single administration. Importantly, such effects have been linked to rapid neuroplastic events, including synaptogenesis; yet, the drug also has effects on multiple neurotransmitter systems and influences immune factors. Other potential emerging treatments include cytokines with trophic properties, such as EPO. Indeed, through its effects on BDNF, immune and neuroplastic processes, EPO holds tremendous possibility as an adjunct treatment that could be co-administered with an SSRI or other standard antidepressant agent. In effect, future pharmacogenic approaches might be utilized to tailor specific treatment combinations to specific individuals with certain genetic polymorphisms and life stressor histories. However, whatever the case may be, we believe that all such treatments will exert and/or maintain positive clinical outcomes, at least in part, by affecting the plasticity of emotional circuits.

## Conflict of interest statement

The authors declare that the research was conducted in the absence of any commercial or financial relationships that could be construed as a potential conflict of interest.
